# Supplementary material for: Experimental investigation of flexible eight-port asymmetric fed MIMO antenna with narrow-super-widebandn-s characteristics for future applications including internet of things
Source: PLoS One. 2025 May 14;20(5):e0322279. doi: 10.1371/journal.pone.0322279 (PMC12077693; doi:10.1371/journal.pone.0322279)
Supplement: Attachment — Submitted filename: Review Comments.docx [file pone.0322279.s011.docx]

**Review Comments**

**For**

“**Experimental Investigation of Flexible Eight-Port Asymmetric Fed MIMO Antenna with Narrow-Super-Widebandn-s Characteristics for Future Applications Including Internet of Things**”

The proposed work presents an eight-port MIMO antenna exhibiting super wideband characteristics for wearable applications. The design methodologies have been explained with simulated and measured results for the proposed antenna. However, below are certain suggestions for further improvement of the proposed work:

1. A detailed explanation of the antenna design equations [1]-[4] could be included. An addition of this explanation will improve the reader's understanding of the design process.
2. Please include a justification for the selection of the resonant frequency, *f*_r_, as 20 GHz, considering that the antenna is super wideband.
3. The dimensions of the MIMO antenna (*E*_1_-*E*_6_) depicted in Fig. 6(a) and (b) could also be included in Table. 1.
4. Please consider fragmenting Fig. 6 into multiple figures for improved readability. The diagrams and fabricated prototype could be grouped in one figure, while the results could be presented separately.
5. The decoupling structure can be explained more explicitly highlighting its importance in achieving desired MIMO characteristics.
