# Supplementary material for: Experimental investigation of flexible eight-port asymmetric fed MIMO antenna with narrow-super-widebandn-s characteristics for future applications including internet of things
Source: PLoS One. 2025 May 14;20(5):e0322279. doi: 10.1371/journal.pone.0322279 (PMC12077693; doi:10.1371/journal.pone.0322279)
Supplement: Attachment — Submitted filename: Review Comments_2.docx [file pone.0322279.s003.docx]

**Review Comments**

**For**

“**Experimental Investigation of Flexible Eight-Port Asymmetric Fed MIMO Antenna with Narrow-Super-Widebandn-s Characteristics for Future Applications Including Internet of Things**”

The authors have addressed all my queries satisfactorily, and the revisions have significantly improved the quality of the manuscript. In my opinion, the paper is now suitable for publication.
